# Cutaneous Manifestations and Hormonal Changes Among Polycystic Ovary Syndrome Patients at a Tertiary Care Center

**DOI:** 10.7759/cureus.20593

**Published:** 2021-12-22

**Authors:** Yara E Aljefri, Rana A Alahmadi, Rakan S Alajmi, Taif A Alkhamisi, Hadeel A Maaddawi, Ali A Alraddadi, Awadh M Alamri

**Affiliations:** 1 College of Medicine, King Saud Bin Abdulaziz University for Health Sciences, Jeddah, SAU; 2 College of Medicine, King Abdullah International Medical Research Center, Jeddah, SAU; 3 Medicine, King Saud Bin Abdulaziz University for Health Sciences College of Medicine, Jeddah, SAU; 4 Department of Dermatology, King Abdulaziz Medical City, Western Region, Jeddah, SAU

**Keywords:** referral pattern, medical comorbidities, hormone levels, cutaneous manifestations, polycystic ovary syndrome (pcos)

## Abstract

Background: Polycystic ovary syndrome (PCOS) is a highly prevalent endocrine disorder affecting 5%-10% of women worldwide. PCOS patients usually present with cutaneous manifestations of hyperandrogenism, such as acne, hirsutism, and androgenic alopecia.

Objective: To estimate the prevalence of dermatological manifestations and their association with hormonal changes in PCOS patients. In addition, this study aimed to estimate the prevalence of comorbidities associated with PCOS and to demonstrate the referral pattern among Dermatology, Gynecology, and Primary Health Care (PHC).

Methods: This is a cross-sectional study conducted at King Abdulaziz Medical City (KAMC) in Jeddah, Saudi Arabia. All PCOS patients who attended KAMC from 2016 to 2021 were included. Data were collected through a retrospective chart review of the electronic medical record system (BestCare) and by utilizing a structured data collection sheet.

Results: A total of 447 female patients were diagnosed with PCOS with a median age of 29 years and a median BMI of 28.76 kg/m^2^.The prevalence of cutaneous manifestations among patients was 68%. Hirsutism (47.3%), acne vulgaris (40.6%), and androgenic alopecia (20.3%) were the most common manifestations. The most common hormonal abnormalities were raised luteinizing hormone (LH) levels in 220 (49.1%) patients and raised LH/follicle-stimulating hormone (FSH) ratio in 159 (35.5%) patients. FSH, LH/FSH ratio, and age were significant predictors for acne vulgaris (P-value=0.01, 0.04, and 0.01, respectively). Obesity (44.20%), infertility (25.70%), and dyslipidemia (17%) were the most common comorbidities in our sample. Most patients' first visits and follow-ups were in PHC.

Conclusion: The prevalence of cutaneous manifestations among PCOS patients is relatively high and plays a significant role in making the diagnosis. Therefore, physicians across multiple specialties need to be more aware of the full spectrum of PCOS presentations to alleviate it from its under-diagnosed status.

## Introduction

Polycystic ovary syndrome (PCOS) is a highly prevalent heterogeneous endocrine disorder that affects 5%-10% of reproductive-aged women worldwide [1]. The diagnosis of PCOS is currently established by the 2003 Rotterdam Consensus criteria. After the exclusion of other disorders that cause excess androgen and menstrual disturbances, this criterion is defined by the presence of at least two out of the three following findings: (a) oligomenorrhea or amenorrhea, (b) clinical signs or biochemical evidence of hyperandrogenism (HA), and (c) polycystic ovaries (≥12 follicles in each ovary measuring 2-9 mm in diameter and/or increased ovarian volume >10 mL) as demonstrated by transvaginal ultrasonography [2]. The hallmark of PCOS is disrupted hypothalamic-pituitary-ovarian axis that leads to high pulse frequency gonadotropin-releasing hormone (GnRH) secretion, which favors luteinizing hormone (LH) secretion over follicle-stimulating hormone (FSH) secretion. The subsequently high LH/FSH ratio stimulates the ovarian theca cells to synthesize excess androgen causing hyperandrogenemia and clinical symptoms of hyperandrogenism [3]. The endocrine profile of women with PCOS includes high levels of adrenal and ovarian androgen and estrogen, abnormalities in gonadotropin (LH and FSH) levels, low levels of serum sex hormone-binding globulin (SHBG), and sometimes elevated serum insulin [4].

Women with PCOS often present with cutaneous symptoms of hyperandrogenism. Therefore, dermatologists have a primary role in the early diagnosis of the disease [5]. Hirsutism is the most common clinical indicator of hyperandrogenism. Other less common skin manifestations of excess androgen include acne vulgaris, androgenic alopecia, oily skin, and seborrhea [6]. In addition, PCOS patients may have acanthosis nigricans as a sign of insulin resistance, a state that has been found to enhance androgen secretion, inhibit the hepatic synthesis of SHBG, and increase total testosterone levels. By this mechanism, obese women with PCOS have a more severe form of hyperandrogenism, hirsutism, and acne [7]. In addition to obesity, PCOS increases the risk of certain medical conditions like type II diabetes mellitus, hypertension, dyslipidemia, cardiovascular disease (CVD), menstrual disturbances, and infertility [8]. As the changes in the physical appearance of PCOS patients accumulate and intensify, they result in psychosocial manifestations like anxiety, depression, and eating disorders [9]. Early detection and management of PCOS are important to prevent its long-term complications and psychosocial effects.

Studies exploring cutaneous manifestations and their association with hormonal changes were performed in various countries [10-12]. However, in Saudi Arabia, this association has not been explored yet. Only a few studies conducted on PCOS have discussed the hormonal, clinical, and ultrasound profiles [13-15]. This retrospective chart review, compelled by the lack of comprehensive reports, aimed to define the prevalence of dermatological manifestations in Saudi PCOS patients and to determine the association these manifestations have with hormonal changes. Additionally, this study aimed to identify the patients’ most common comorbidities, and their pattern of referral and follow-up with Dermatology, Gynecology, and Primary Health Care (PHC) in a tertiary care center.

## Materials and methods

This is a cross-sectional study conducted at King Abdulaziz Medical City (KAMC) in Jeddah, Saudi Arabia. The study included all PCOS female patients who attended Dermatology clinic, Obstetrics and Gynecology clinic, and PHC from May 2016 to October 2021. Data were collected through a retrospective chart review of the electronic medical record system (BestCare) and by utilizing a structured data collection sheet. The data collection sheet consisted of socio-demographic variables like age and body mass index (BMI). Clinical variables like cutaneous manifestations, hormonal profile, and comorbidities of the patients were included in addition to patients’ referral patterns among dermatologists, gynecologists, and PHC physicians. Patients were considered to have abnormally high or low levels of a hormone if their records revealed results out of the following ranges: prolactin (7.20-44.30) µg/L, testosterone (0.5-4.2) nmol/L, thyroid-stimulating hormone (TSH) (0.60-5.00) mlU/L, hemoglobin A1c (HbA1c) (3.9%-6.1%), fasting blood glucose (3.9-5.8) mmol/L, LH (2.39-6.60) IU/L, FSH (3.4-21.6) IU/L, and LH/FSH ratio more or less than 1:1. The obtained data from the sheet were entered into Microsoft Excel 2016 (Microsoft Corporation, Redmond, WA, USA) then entered and analyzed in IBM Statistical Package for the social sciences (SPSS) version 25.0 (IBM Corp., Armonk, NY, USA). Descriptive statistics were used to present categorical variables in frequencies and percentages, and continuous variables were presented as the median and interquartile range (IQR). Binary logistic regression was used to determine whether the age or hormone levels are predictors for the incidence of cutaneous manifestations. A P-value that is less than 0.05 was considered to be significant. All patients’ data were secured, and ethical approval was received from the Institutional Review Board (IRB) at King Abdullah International Medical Research Centre, National Guard Health Affairs (NGHA), Jeddah, Saudi Arabia (Reference number: JED-21-427780-135840) in accordance with The Code of Ethics of the World Medical Association (Declaration of Helsinki).

## Results

The present study included 447 female patients who were diagnosed with PCOS. Patients' ages ranged from 15 to 55 years old. The median age was 29 years. The median BMI was 28.76 kg/m^2^, which was interpreted as being overweight. Patients' first visits, when the diagnosis was made, and follow-ups were reported for 350 patients. The first visit was to PHC for 234 patients (52.2%), Gynecology for 75 patients (16.7%), and Dermatology for 41 patients (9.2%). The referral pattern and follow-up visits with PHC, Gynecology, and Dermatology have shown the same order with the following percentages (60.3%), (9.2%), and (8.5%), respectively. The details are shown in Table 1.

**Table 1:** Patients' demographics BMI: Body mass index, IQR: Interquartile range

Variable	N=447
Age, median (IQR)	29 (25-34)
BMI, median (IQR)	28.76 (24.49-34.56)
Referral pattern, n (%)	
First visit	
Primary health care	234 (52.2%)
Gynecology	75 (16.7%)
Dermatology	41 (9.2%)
Referral to/follow up with	
Primary health care	270 (60.3%)
Gynecology	41 (9.2%)
Dermatology	38 (8.5%)

Examination in the outpatient setting reported 304 (68%) patients with at least one dermatological manifestation. Hirsutism, acne vulgaris, and androgenic alopecia were the most common cutaneous symptoms observed in 212 (47.3%), 182 (40.6%), and 91 (20.3%) patients, respectively. Post-inflammatory hyperpigmentation (PIH) in the face, cheeks, trunk, extremities, back, elbow, or knees was present in 39 patients. Hair thinning and skin tags were the least reported symptoms, as found in 6 (1.3%) and 5 (1.1%) of patients, respectively. Further details on cutaneous manifestations are given in Table 2.

**Table 2:** Cutaneous manifestations

	N	Percentage
Patients with ≥ 1 cutaneous manifestation	304	68%
Patients without any cutaneous manifestation	143	32%
Hirsutism	212	47.3%
Acne Vulgaris	182	40.6%
Hair loss/androgenic alopecia	91	20.3%
Post-inflammatory Hyperpigmentation	39	8.7%
Atopic dermatitis	26	5.8%
Acanthosis Nigricans	23	5.1%
Folliculitis	12	2.7%
Hair thinning	6	1.3%
Skin tag	5	1.1%

The most common hormonal abnormalities encountered in our patients were raised LH levels in 220 (49.1%) patients and raised LH/FSH ratio in 159 (35.5%) patients. A great number of patients had normal levels of TSH (82.6%), prolactin (72.1%), FSH (71.4%), testosterone (58.5%), glycosylated hemoglobin (HbA1c) (55.8%), and fasting blood glucose (55.4%). Table 3 illustrates further details on hormone levels.

**Table 3:** Hormone levels LH: Luteinizing hormone, FSH: Follicle-stimulating hormone, TSH: Thyroid-stimulating hormone, HbA1c: Hemoglobin A1c

LH (IU/L)	
Median (IQR)	7.18 (4.32-11.39)
Abnormally high, n (%)	220 (49.1%)
Normal, n (%)	140 (31.3%)
Abnormally low, n (%)	51 (11.4%)
LH/FSH ratio	
Median (IQR)	1.66 (1.01-2.63)
Abnormally high, n (%)	159 (35.5%)
Normal, n (%)	150 (33.5%)
Abnormally low, n (%)	99 (22.1%)
Fasting blood glucose (nmol/L)	
Median (IQR)	4.90 (4.60-5.40)
Abnormally high, n (%)	39 (8.7%)
Normal, n (%)	248 (55.4%)
Abnormally low, n (%)	5 (1.1%)
TSH (mlU/L)	
Median (IQR)	1.88 (1.16-2.90)
Abnormally high, n (%)	31 (6.9%)
Normal, n (%)	370 (82.6%)
Abnormally low, n (%)	28 (6.3%)
Prolactin (ug/L)	
Median (IQR)	13.03 (8.73-19.49)
Abnormally high, n (%)	23 (5.1%)
Normal, n (%)	323 (72.1%)
Abnormally low, n (%)	59 (13.2%)
HbA1c (%)	
Median (IQR)	5.30 (5.00-5.60)
Abnormally high, n (%)	22 (4.9%)
Normal, n (%)	250 (55.8%)
Abnormally low, n (%)	3 (0.7%)
FSH (IU/L)	
Median (IQR)	4.57 (3.58-5.53)
Abnormally high, n (%)	4 (0.9%)
Normal, n (%)	320 (71.4%)
Abnormally low, n (%)	92 (20.5%)
Testosterone (nmol/L)	
Median (IQR)	1.70 (1.22-2.20)
Abnormally high, n (%)	3 (0.7%)
Normal, n (%)	262 (58.5%)
Abnormally low, n (%)	5 (1.1%)

FSH, LH/FSH ratio, and age were significant predictors for acne vulgaris (P-value=0.01, 0.04, and 0.01, respectively). Table 4 summarizes the binary logistic regression results.

**Table 4:** Logistic regression analysis with cutaneous manifestations as dependent variables LH: Luteinizing hormone, FSH: Follicle-stimulating hormone, TSH: Thyroid-stimulating hormone, HbA1c: Hemoglobin A1c

	Odds ratio (95% CI)	P-value
Acne Vulgaris		
LH	1.121 (0.96-1.32)	0.16
FSH	0.53 (0.32-0.86)	0.01
LH/FSH ratio	0.43 (0.19-0.95)	0.04
TSH	0.85 (0.64-1.13)	0.26
Testosterone	1.22 (0.73-2.04)	0.44
Prolactin	1.01 (0.99-1.03)	0.28
HbA1c	0.75 (0.43-1.31)	0.32
Fasting blood glucose	0.89 (0.63-1.25)	0.50
Age	0.92 (0.86-0.98)	0.01
Hirsutism		
LH	0.95 (0.88-1.02)	0.14
FSH	0.94 (0.72-1.24)	0.66
LH/FSH ratio	1.06 (0.84-1.32)	0.63
TSH	1.02 (0.78-1.32)	0.90
Testosterone	1.51 (0.95-2.40)	0.08
Prolactin	1.00 (0.99-1.02)	0.68
HbA1c	0.74 (0.47-1.17)	0.20
Fasting blood glucose	1.03 (0.80-1.32)	0.82
Age	0.98 (0.93-1.05)	0.61
Acanthosis Nigricans		
LH	1.06 (0.81-1.38)	0.70
FSH	0.81 (0.38-1.70)	0.58
LH/FSH ratio	0.66 (0.17-2.47)	0.53
TSH	0.76 (0.43-1.34)	0.35
Testosterone	1.60 (0.68-3.76)	0.28
Prolactin	0.97 (0.91-1.03)	0.29
HbA1c	0.92 (0.49-1.73)	0.80
Fasting blood glucose	0.46 (0.19-1.12)	0.09
Age	0.97 (0.87-1.09)	0.65
Hair loss/androgenic alopecia		
LH	1.07 (0.91-1.26)	0.44
FSH	0.77 (0.46-1.29)	0.32
LH/FSH ratio	1.23 (0.56-2.68)	0.60
TSH	0.90 (0.65-1.24)	0.51
Testosterone	0.87 (0.49-1.55)	0.64
Prolactin	1.00 (0.98-1.03)	0.75
HbA1c	0.70 (0.41-1.18)	0.18
Fasting blood glucose	1.19 (0.91-1.55)	0.21
Age	0.97 (0.90-1.04)	0.42
Hair thinning		
LH	0.98 (0.79-1.21)	0.86
FSH	1.02 (0.47-2.21)	0.97
LH/FSH ratio	0.96 (0.72-1.28)	0.77
TSH	0.80 (0.37-1.71)	0.56
Testosterone	0.14 (0.02-1.30)	0.08
Prolactin	1.02 (0.96-1.07)	0.58
HbA1c	0.70 (0.41-1.18)	0.79
Fasting blood glucose	1.57 (0.92-2.66)	0.10
Age	0.99 (0.82-1.20)	0.92
Folliculitis		
LH	0.88 (0.53-1.48)	0.64
FSH	0.78 (0.29-2.06)	0.61
LH/FSH ratio	0.77 (0.13-4.61)	0.77
TSH	1.05 (0.61-1.80)	0.86
Testosterone	0.30 (0.06-1.58)	0.16
Prolactin	1.00 (0.94-1.06)	0.87
HbA1c	1.26 (0.30-5.34)	0.76
Fasting blood glucose	1.07 (0.56-2.06)	0.83
Age	1.00 (0.87-1.15)	1.00
Skin tag		
LH	1.09 (0.49-2.45)	0.83
FSH	1.63 (0.33-8.06)	0.55
LH/FSH ratio	0.16 (0.00-93.73)	0.57
TSH	0.78 (0.22-2.83)	0.71
Testosterone	0.98 (0.16-5.87)	0.98
Prolactin	0.97 (0.88-1.08)	0.59
HbA1c	0.51 (0.09-2.92)	0.45
Fasting blood glucose	0.27 (0.03-2.44)	0.24
Age	1.13 (0.97-1.32)	0.11
Atopic dermatitis		
LH	0.95 (0.80-1.13)	0.55
FSH	0.90 (0.50-1.64)	0.74
LH/FSH ratio	1.12 (0.73-1.73)	0.59
TSH	1.24 (0.76-2.00)	0.39
Testosterone	0.89 (0.34-2.38)	0.82
Prolactin	0.93 (0.84-1.03)	0.14
HbA1c	0.61 (0.25-1.49)	0.28
Fasting blood glucose	0.92 (0.46-1.85)	0.82
Age	0.92 (0.80-1.06)	0.24
Post-inflammatory Hyperpigmentation		
LH	0.92 (0.81-1.05)	0.23
FSH	1.12 (0.76-1.64)	0.56
LH/FSH ratio	0.96 (0.71-1.29)	0.77
TSH	1.15 (0.82-1.61)	0.41
Testosterone	1.10 (0.59-2.02)	0.77
Prolactin	1.01 (0.98-1.03)	0.68
HbA1c	0.67 (0.36-1.25)	0.21
Fasting blood glucose	0.65 (0.30-1.38)	0.26
Age	1.05 (0.97-1.13)	0.21

The most reported comorbidities were obesity (44.20%), infertility (25.70%), and dyslipidemia (17%). On the other hand, CVD was the least reported comorbidity in 12 (2.70%) patients. Other comorbidities, in order of prevalence, are shown in Table 5.

**Table 5:** Patients' comorbidities

	N	Percentage
Obesity	198	44.20%
Infertility	115	25.70%
Dyslipidemia	76	17%
Migraine or headache	40	8.90%
Diabetes mellitus	34	7.60%
Psychological disorder	29	6.50%
Hypertension	25	5.60%
Asthma	22	4.90%
Cardiovascular comorbidities	12	2.70%

## Discussion

We aimed in this study to define the cutaneous manifestations of PCOS in the Saudi population and examine the relationship these manifestations have with the hormonal changes of PCOS. Our results demonstrated a high prevalence of cutaneous manifestations, the most common being hirsutism, acne vulgaris, and androgenic alopecia. The hormonal profile of our sample showed an abnormally high LH in approximately half, LH/FSH ratio that was high, normal, and low in equal thirds of our sample, and FSH that was abnormally low for 20.5%. Most patients had normal levels of TSH, prolactin, testosterone, HbA1c, and fasting blood glucose. Acne was negatively associated with FSH, LH/FSH ratio, and age. No other associations were found. Comorbidities afflicting our patients were most commonly obesity, infertility, and dyslipidemia. Most patients were first seen and later followed up in PHC. Gynecology first visit or referral came second and lastly was Dermatology.

Cutaneous manifestations in our patients were commonly in the form of hirsutism (47.3%), closely followed by acne (40.6%). This is similar to a Saudi report by Guraya et al., whose sample showed a 36.1% prevalence of hirsutism and 31.4% of acne [14]. Asdaq et al. reported a much greater difference between the two presentations in their Saudi PCOS patients (84.1% with hirsutism vs 36.6% with acne) [16]. Differences in the prevalence of hirsutism and acne may be due to the different etiologies underlying the two. Hirsutism appears to be closely linked with hyperandrogenism [17-19], and acne more closely with insulin resistance than with androgen excess [20]. In sharp contrast, however, were the recent findings of Abusailik et al. In their study that included 146 Jordanian women with PCOS, the authors found that patients with an abnormal BMI were more likely to suffer from cutaneous manifestations than those with a normal BMI, but the only exception was acne vulgaris. Acne prevalence did not significantly differ between patients with a normal BMI and patients with an abnormal BMI. They also found that patients with acne were more likely to have abnormally high total testosterone, although the association reported was not significant [21]. Androgenic alopecia prevalence in our sample resembles the 20%-30% reported in a systematic review and meta-analysis conducted by Carmina et al. to understand and manage female pattern hair loss in PCOS [22]. Finally, acanthosis nigricans and skin tags were much more prevalent in Keen et al.’s report, at 30% and 9%, respectively [10], than in our sample. This difference may be attributed to differences in our samples’ fasting blood glucose and fasting insulin levels, which serve as reflections of insulin resistance, the pathophysiology responsible for acanthosis nigricans and skin tags [23].

The hormonal profile of our patients showed abnormally high LH in 49.1% and abnormally high LH/FSH ratio in 35.5%, making them the most prevalent hormonal abnormalities. In a previous cross-sectional study, Mulhim et al. uncovered an LH mean that was higher in the PCOS group compared to the control group (8.9 vs 6.03 mIU/mL), and an FSH that was significantly lower (4.9 vs 6.6 mIU/mL) [13]. On the other hand, Alsibyani et al. reported no significant increase in LH, but an abnormally low FSH [15]. All three reports, including ours, showed an LH/FSH ratio that is consistently high in PCOS. Normal TSH levels were observed in the majority of our sample. Similarly, Trummer et al. observed elevated TSH levels in 5.8% of their sample of 583 PCOS women. The authors additionally found an association between TSH levels and a more adverse metabolic profile, which offers an explanation for the low prevalence of abnormal TSH in our sample [24]. Our low prolactin results are consistent with the results of Yang et al., who found significantly lower prolactin levels in PCOS patients compared to non-PCOS controls [25]. On the other hand, Szosland et al. compared prolactin levels of PCOS and non-PCOS patients at nine different timings and found no significant difference between the two groups [26]. Many studies reported the presence of hyperprolactinemia in their PCOS patients to varying extents with no agreed-upon explanation [10,11,13]. Regarding testosterone levels, Keen et al. reported a much greater prevalence (28%) of raised testosterone and was, therefore, the most prevalent abnormality in their sample [10]. Likewise, Mulhim et al. reported significantly higher testosterone mean in PCOS patients compared to controls [13]. The difference in testosterone levels in our samples may be due to unreported differences in the metabolic profiles of our samples, an association observed in a paper by Lerchbaum et al. [27]. In their study of 706 PCOS patients, where they found a significant association between levels of free testosterone and a more adverse metabolic profile on the other side, although more studies are needed to confirm this link.

Evidence answering the question of what hormonal predictors exist for the development of cutaneous manifestations is inconclusive [18,19,28]. Ozdemir et al. found acne to be associated with free testosterone and DHEAS in their prospective investigation of 115 PCOS patients [18]. Similarly, Gowri et al. reported acne’s association with testosterone in 40 patients with PCOS [11]. Hirsutism in our patients was not associated with any hormonal changes, although many papers noted its association with elevated serum testosterone [17,19] and abnormal lipid and glucose profiles [18,19]. Similar to our results of no testosterone association with any of the cutaneous symptoms, Li et al. found that despite nearly equivalent testosterone levels, adolescent girls with PCOS had a significantly higher prevalence of acne and hirsutism compared to adult women with PCOS [28]. In contrast to our finding of no predictors for acanthosis nigricans, Schimdt et al. found acanthosis nigricans to be associated with elevated testosterone, increased obesity, insulin resistance, and dyslipidemia [19]. Androgenic alopecia is often linked to metabolic syndrome [23]. In an interesting, observational study of 2028 Koreans, Kim et al. observed an association between androgenic alopecia and waist circumference, hypertension, and diabetes mellitus. Interestingly, females with androgenic alopecia were more likely to have the cerebrovascular disease, obesity, and dyslipidemia compared to males with androgenic alopecia [29]. Our study however showed no association between androgenic alopecia and measured fasting blood glucose or HbA1c, and our investigation of its correlation with other hormones also yielded no significant associations.

Our study has found obesity to be the most prevalent among comorbidities (44.2%), consistent with Guraya et al. (53.7%) [14]. According to a systematic review and meta-analysis by de Groot et al., obesity is a strong predictor for hypertension in women with PCOS, and PCOS women are at a much higher risk for cardiovascular events, even after adjusting for BMI [30], highlighting the relatively sound metabolic profile our patients showed. The state of dyslipidemia and diabetes mellitus in our study was similar to that of Ehrmann’s [31]. In their study of the prevalence and predictors of metabolic syndrome in PCOS women, Ehrmann et al. found a prevalence of 32% and 6.6% for dyslipidemia and diabetes mellitus, respectively. The nearly double prevalence of dyslipidemia in their sample compared to ours may be explained by the much higher prevalence of obesity (above 85%) in their sample. Infertility was reported in 25.70% of our sample, mirroring the 20.77% reported by Alsibyani et al. [15]. Psychological disorders reported in our patients were at much lower rates than those reported in the literature [16,32]. Asdaq et al. found a difference of 22.4% in the prevalence of depression, 20% of anxiety, and 46.6% of overall stress between PCOS and non-PCOS college students, stressing the psychological burden of PCOS [16]. Another study by Chaudhuri et al. looked at 70 PCOS women aged 18-45 and found a total prevalence of 38.6% for anxiety and 25.7% for depression. Interestingly, they uncovered a possible link between cutaneous manifestations and psychiatric diagnosis, with anxiety patients being more likely to struggle with infertility and alopecia, and depression patients with acne [32]. Asthma was found in 4.9% of our patients, compared to 22.4% in Zierau’s study on the prevalence and severity of asthma in PCOS patients [33]. The co-existence of asthma and PCOS was argued in a previous review by Zierau et al. to be due to shared risk factors such as obesity and systemic inflammation [34].

The majority of our patients first presented to PHC, followed by Gynecology, and Dermatology. The majority were referred to and/or later followed up with PHC. Hardly any paper has analyzed the pattern of visits and referrals among PCOS patients, though a paper by Sivayoganathan et al. may offer some insight. In their paper, the authors sought PCOS patients referred from PHC to Infertility, Gynecology, Dermatology, and Endocrine clinics, and their attempt to compare the different presentations of PCOS produced a significant finding. PCOS women not yet diagnosed were mostly those referred to Dermatology clinics (i.e., had dermatological complaints) and to endocrine clinics (i.e., had weight problems) [5]. Indeed, physicians across multiple specialties need to be more conscious of the full spectrum of PCOS presentations.

To the best of our knowledge, this study was the first to attempt to draw associations between the individual hormonal changes and cutaneous manifestations of PCOS in the Saudi population, as well as illustrate the pattern of presentation and referral for PCOS patients in Saudi Arabia. Additional is the reporting of certain manifestations such as PIH, folliculitis, and atopic dermatitis in Saudi PCOS patients.

Our study was limited by the lack of a control group to compare with our PCOS patients. The retrospective design has also limited our ability to account for confounders. Moreover, a better, more comprehensive investigation of the hormonal profile of our sample would have made a comparison with other papers more feasible. We recommend future studies adopt a prospective design for a better understanding of how the different cutaneous and hormonal parameters of PCOS are connected. More homogenous samples and sets of variables would also be of help in comparing the evidence.

## Conclusions

The overall prevalence of skin manifestations among PCOS patients was 68%. The most prevalent manifestations were hirsutism followed by acne vulgaris and androgenic alopecia. The most common hormonal abnormalities were raised LH levels and raised LH/FSH ratio. Age, FSH, and LH/FSH ratio were significant predictors for acne vulgaris. The most common comorbid conditions were obesity, infertility, and dyslipidemia. In addition, the referral pattern and follow-up visits for most PCOS patients were in PHC then Gynecology and Dermatology. Further multicenter studies are warranted to clarify the prevalence of skin manifestations and hormonal association among PCOS patients in Saudi Arabia in further detail.

## References

[1] Patel S (2018). Polycystic ovary syndrome (PCOS), an inflammatory, systemic, lifestyle endocrinopathy. J Steroid Biochem Mol Biol.

[2] Dumesic DA, Oberfield SE, Stener-Victorin E, Marshall JC, Laven JS, Legro RS (2015). Scientific statement on the diagnostic criteria, epidemiology, pathophysiology, and molecular genetics of polycystic ovary syndrome. Endocr Rev.

[3] Fauser BC, Pache TD, Lamberts SW, Hop WC, de Jong FH, Dahl KD (1991). Serum bioactive and immunoreactive luteinizing hormone and follicle-stimulating hormone levels in women with cycle abnormalities, with or without polycystic ovarian disease. J Clin Endocrinol Metab.

[4] Lause M, Kamboj A, Fernandez Faith E (2017). Dermatologic manifestations of endocrine disorders. Transl Pediatr.

[5] Sivayoganathan D, Maruthini D, Glanville JM, Balen AH (2011). Full investigation of patients with polycystic ovary syndrome (PCOS) presenting to four different clinical specialties reveals significant differences and undiagnosed morbidity. Hum Fertil (Camb).

[6] Chuan SS, Chang RJ (2010). Polycystic ovary syndrome and acne. Skin Therapy Lett.

[7] Moura HH, Costa DL, Bagatin E, Sodré CT, Manela-Azulay M (2011). Polycystic ovary syndrome: a dermatologic approach. An Bras Dermatol.

[8] Zeng X, Xie YJ, Liu YT, Long SL, Mo ZC (2020). Polycystic ovarian syndrome: correlation between hyperandrogenism, insulin resistance and obesity. Clin Chim Acta.

[9] Cooney LG, Lee I, Sammel MD, Dokras A (2017). High prevalence of moderate and severe depressive and anxiety symptoms in polycystic ovary syndrome: a systematic review and meta-analysis. Hum Reprod.

[10] Keen MA, Shah IH, Sheikh G (2017). Cutaneous manifestations of polycystic ovary syndrome: a cross-sectional clinical study. Indian Dermatol Online J.

[11] Gowri BV, Chandravathi PL, Sindhu PS, Naidu KS (2015). Correlation of skin changes with hormonal changes in polycystic ovarian syndrome: a cross-sectional study clinical study. Indian J Dermatol.

[12] Kaur S, Gupta S, Juneja S (2020). Study of cutaneous manifestations of polycystic ovarian syndrome. Int J Reprod Contracept Obstet Gynecol.

[13] Al-Mulhim AA, Abul-Heija AA, Al-Talib AA (2013). Hormonal, metabolic and clinical profile of Saudi women with polycystic ovary syndrome. Saudi J Med Med Sci.

[14] Guraya SS (2013). Prevalence and ultrasound features of polycystic ovaries in young unmarried Saudi females. J Microsc Ultrastruct.

[15] Alsibyani NA, Malibary MA, Derham AA (2018). Clinical presentation of polycystic ovary syndrome among Saudi Arabian women-Jeddah-Saudi Arabia. J Hosp Med.

[16] Asdaq SM, Yasmin F (2020). Risk of psychological burden in polycystic ovary syndrome: a case control study in Riyadh, Saudi Arabia. J Affect Disord.

[17] Hong JS, Kwon HH, Park SY (2015). Cutaneous manifestations of the subtypes of polycystic ovary syndrome in Korean patients. J Eur Acad Dermatol Venereol.

[18] Ozdemir S, Ozdemir M, Görkemli H, Kiyici A, Bodur S (2010). Specific dermatologic features of the polycystic ovary syndrome and its association with biochemical markers of the metabolic syndrome and hyperandrogenism. Acta Obstet Gynecol Scand.

[19] Schmidt TH, Khanijow K, Cedars MI (2016). Cutaneous findings and systemic associations in women with polycystic ovary syndrome. JAMA Dermatol.

[20] Kartal D, Yildiz H, Ertas R, Borlu M, Utas S (2016). Association between isolated female acne and insulin resistance: a prospective study. G Ital Dermatol Venereol.

[21] Abusailik MA, Muhanna AM, Almuhisen AA, Alhasanat AM, Alshamaseen AM, Mustafa SMB, Nawaiseh MB (2021). Cutaneous manifestation of polycystic ovary syndrome. Dermatol Reports.

[22] Carmina E, Azziz R, Bergfeld W (2019). Female pattern hair loss and androgen excess: a report from the Multidisciplinary Androgen Excess and PCOS Committee. J Clin Endocrinol Metab.

[23] Misitzis A, Cunha PR, Kroumpouzos G (2019). Skin disease related to metabolic syndrome in women. Int J Womens Dermatol.

[24] Trummer C, Schwetz V, Giuliani A, Obermayer-Pietsch B, Lerchbaum E (2015). Impact of elevated thyroid-stimulating hormone levels in polycystic ovary syndrome. Gynecol Endocrinol.

[25] Yang H, Di J, Pan J, Yu R, Teng Y, Cai Z, Deng X (2020). The association between prolactin and metabolic parameters in PCOS women: a retrospective analysis. Front Endocrinol (Lausanne).

[26] Szosland K, Pawlowicz P, Lewiński A (2015). Prolactin secretion in polycystic ovary syndrome (PCOS). Neuro Endocrinol Lett.

[27] Lerchbaum E, Schwetz V, Rabe T, Giuliani A, Obermayer-Pietsch B (2014). Hyperandrogenemia in polycystic ovary syndrome: exploration of the role of free testosterone and androstenedione in metabolic phenotype. PLoS One.

[28] Li L, Yang D, Chen X, Chen Y, Feng S, Wang L (2007). Clinical and metabolic features of polycystic ovary syndrome. Int J Gynaecol Obstet.

[29] Kim BK, Choe SJ, Chung HC, Oh SS, Lee WS (2018). Gender-specific risk factors for androgenetic alopecia in the Korean general population: associations with medical comorbidities and general health behaviors. Int J Dermatol.

[30] de Groot PC, Dekkers OM, Romijn JA, Dieben SW, Helmerhorst FM (2011). PCOS, coronary heart disease, stroke and the influence of obesity: a systematic review and meta-analysis. Hum Reprod Update.

[31] Ehrmann DA, Liljenquist DR, Kasza K, Azziz R, Legro RS, Ghazzi MN (2006). Prevalence and predictors of the metabolic syndrome in women with polycystic ovary syndrome. J Clin Endocrinol Metab.

[32] Chaudhari AP, Mazumdar K, Mehta PD (2018). Anxiety, depression, and quality of life in women with polycystic ovarian syndrome. Indian J Psychol Med.

[33] Zierau L, Cortes R, Thomsen SF, Jimenez-Solem E, Lindenberg S, Backer V (2018). Asthma severity and fertility outcome in women with polycystic ovary syndrome: a registry-based study. ERJ Open Res.

[34] Zierau L, Gade EJ, Lindenberg S, Backer V, Thomsen SF (2016). Coexistence of asthma and polycystic ovary syndrome: a concise review. Respir Med.

